# Modeling the Impact of MMR Vaccination Strategies on Measles Outbreaks in Texas

**DOI:** 10.1001/jamahealthforum.2025.3992

**Published:** 2025-09-19

**Authors:** Kaiming Bi, Thuy Nguyen, Boya Peng, Trudy Krause, Cecilia Ganduglia Cazaban, Janelle Rios, Cici Bauer, Catherine Troisi, Eric Boerwinkle, Aanand D. Naik

**Affiliations:** 1 School of Public Health, The University of Texas Health Science Center at Houston, Houston; 2 Texas Epidemic Public Health Institute (TEPHI), Houston

## Abstract

This study uses a mathematical model to simulate a hypothetical measles case importation and estimate potential outbreak sizes across various Texas counties.

## Introduction

Measles was a ubiquitous childhood disease in the US until widespread adoption of the measles, mumps, rubella (MMR) vaccine in 1971. This led to a dramatic decline in cases and declaration of measles elimination in 2000.^1^ Periodic outbreaks have since reemerged, driven by lapses in vaccination and the importation of cases from abroad.^2^ On January 29, 2025, local transmission was identified among 4 school-aged children in Gaines County, Texas.^3^ By June 18, 2025, Texas reported 750 measles cases, with 413 occurring in Gaines County alone. These events underscore the challenges in maintaining community immunity in the setting of vaccine hesitancy^4^ and disruption of routine vaccination programs, including drops in MMR coverage.^5^

## Methods

We developed a mathematical model (eMethods in Supplement 1), first calibrated using reported case data from Gaines County and local vaccination rates.^4,6^ Expanding our analysis, we simulated a hypothetical measles case importation on January 20, 2025—coinciding with the introduction of patient zero in Gaines County—to estimate potential outbreak sizes across various Texas counties under 3 scenarios: (1) reported vaccination rates for each county, referred to as baseline; (2) a 5% reduction in reported vaccination coverage; and (3) a 5% increase in reported vaccination coverage for each county. Epidemic dynamics were projected through June 10, 2025. Although this approach facilitated direct scenario comparisons, it assumed independent, nonspatially correlated outbreaks in each county and did not model intercounty transmission.

This research used publicly available, deidentified data and did not involve human participants; therefore, it was determined to be exempt from institutional review board review and did not require approval. This study followed STRESS guidelines for reporting modeling and simulation research.

## Results

**Figure 1.**
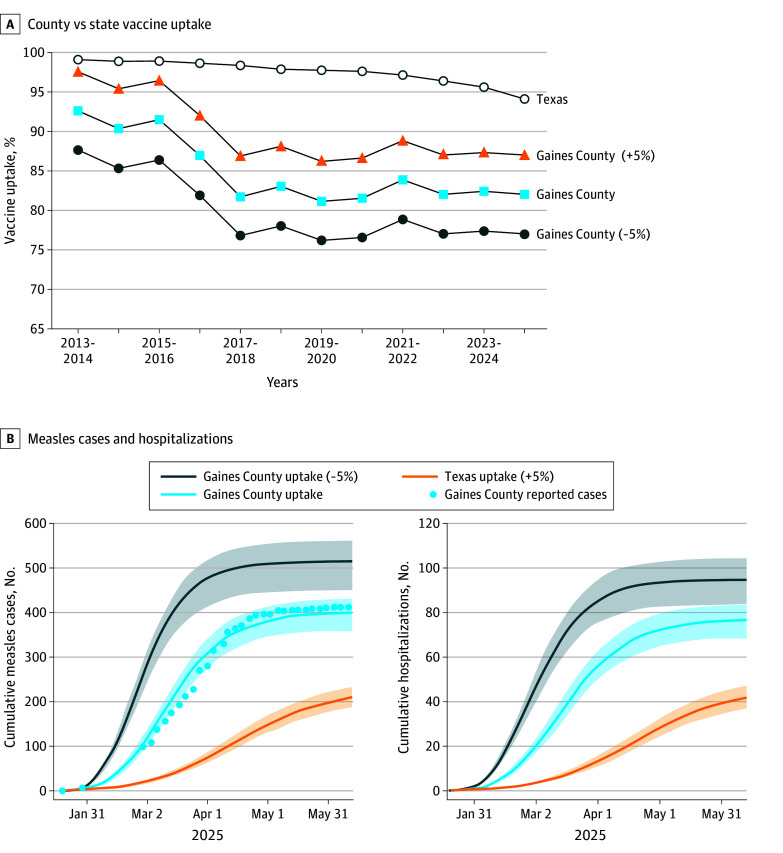
Vaccination Rates and Counterfactual Scenario Modeling Projections for the 2025 Measles Outbreak in Gaines County and Texas Overall A, Vaccination rates for kindergarten students from 2013 to 2025 in Gaines County and Texas. B, Counterfactual scenario projection results for Gaines County. The estimated reported measles cases and hospitalizations are shown for the baseline scenario (light blue), an elevated vaccination scenario assuming vaccine coverage increased by 5% from the reported levels (orange), and a depressed vaccination scenario with coverage reduced by 5% from the reported levels (dark blue). Lines and shaded areas represent the median and 95% estimation intervals across 200 stochastic simulations, respectively.

**Figure 2.**
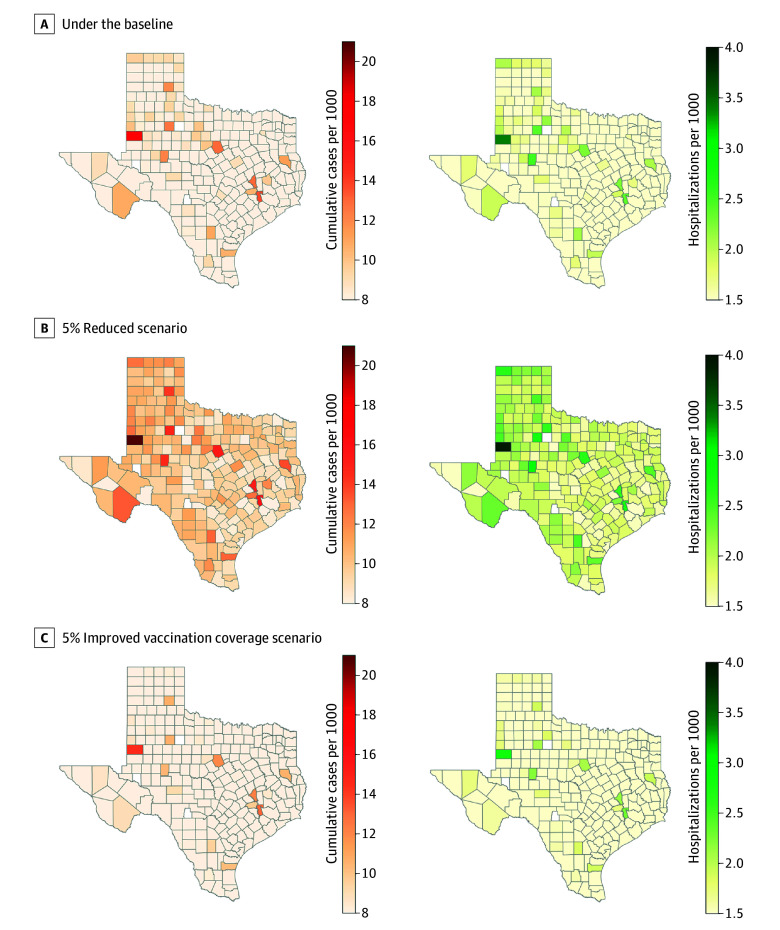
Heatmaps of Cumulative Reported Cases From Simulations Under Baseline, 5% Reduced, and 5% Improved Vaccination Coverage Scenarios Heatmaps of the median cumulative reported cases per 1000 population from the simulations under the baseline (A), 5% reduced (B), and 5% improved (C) vaccination coverage scenarios. These estimates reflect simulated outbreak sizes, assuming independent dynamics within each county, without accounting for spatial correlation or intercounty transmission. The simulations were run from January 20 to June 10, 2025, with median results generated from 200 stochastic simulations.

In Texas, the kindergarten MMR vaccination rates fell from 98.5% in 2013 to 2014 to 94.3% in 2024 to 2025, and in Gaines County, it declined even more, dropping from 92.6% to 82.0% over the same periods (Figure 1A).^6^ Under the baseline model scenario across all Texas counties, Gaines county (Figure 1B), along with Walker (14 [95% CI, 12-15] cases per 1000), Brazos (13 [95% CI, 12-14] cases per 1000), Erath (13 [95% CI, 11-14] cases per 1000), Kent (13 [95% CI, 11-14] cases per 1000), and Sterling (12 [95% CI, 11-13] cases per 1000) counties were estimated to experience outbreaks exceeding 12 cases per 1000 population (Figure 2A).

Under the 5% reduction scenario, outbreak magnitudes increased substantially. Gaines (21 [95% CI, 19-23] cases per 1000; 4 [95% CI, 3-5] hospitalizations per 1000), Walker (16 [95% CI, 14-18] cases per 1000; 3 [95% CI, 2-3] hospitalizations per 1000), Brazos (15 [95% CI, 14-17] cases per 1000; 3 [95% CI, 2-3] hospitalizations per 1000), and Erath (15 [95% CI, 14-17] cases per 1000; 3 [95% CI, 2-3] hospitalizations per 1000) counties were each projected to exceed 15 reported cases and 3 hospitalizations per 1000 population (Figure 2B). In contrast, if all counties increased their current vaccination rates by 5%, no county was expected to exceed 15 reported cases and 3 hospitalizations per 1000 population (Figure 2C).

## Discussion

Findings of this study highlight the critical role of improving MMR vaccination coverage to prevent large-scale measles outbreaks, particularly in regions with declining immunization rates. Projecting potential measles outbreaks that have not yet emerged requires making assumptions about case importation, transmission dynamics, undocumented vaccination rates in certain age groups, and other factors that remain incompletely understood—particularly the complex behaviors of unvaccinated populations during an outbreak. Nonetheless, even if some assumptions are later disproven, simulating a range of carefully constructed scenarios can meaningfully contribute to pandemic preparedness. The present modeling framework is readily adaptable to other locations with sufficient epidemiological and vaccination data beyond Texas.

## Notes

Supplement 1.
eMethods

eReferences

Supplement 2.
Data Sharing Statement
