# Supplementary material for: Modeling the Impact of MMR Vaccination Strategies on Measles Outbreaks in Texas
Source: JAMA Health Forum. 2025 Sep 19;6(9):e253992. doi: 10.1001/jamahealthforum.2025.3992 (PMC12449715; doi:10.1001/jamahealthforum.2025.3992)
Supplement: Supplement 1. — eMethods eReferences [file jamahealthforum-e253992-s001.pdf]

Supplemental Online Content

Bi K, Nguyen T, Peng B, et al. Modeling the impact of MMR vaccination strategies on measles outbreaks in Texas. *JAMA Health Forum*. 2025;6(9):e253992.
doi:10.1001/jamahealthforum.2025.3992

eMethods
eReferences

This supplemental material has been provided by the authors to give readers additional information about their work.

© 2025 Bi K et al. *JAMA Health Forum*.

eMethods

Data

Census Data: Age-specific population data for Texas counties were obtained from the 2023 Annual County and Puerto Rico Municipio Resident Population Estimates by Single Year of Age and Sex, as reported by the United States Census Bureau [7].

Measles Case Data: The case data were obtained from the Texas Department of State Health Services (DSHS) [4]. Beginning February 28, 2025, DSHS issued biweekly measles outbreak briefs every Tuesday and Friday.

Measles Case and Hospitalization age distributions: The CDC reports the age breakdowns for measles cases and hospitalizations, updating the data on a weekly basis [8].

Measles vaccination rates: DSHS provided annual MMR vaccination rates for kindergarten students across all Texas counties from the 2013–2014 to 2024–2025 school years [9][6].

Model Description

We employed an age-structured SEIR (Susceptible-Exposed-Infectious-Recovered) model to simulate measles transmission dynamics, incorporating health outcomes such as hospitalizations and deaths (**Figure S.1**). Vaccination efficacy was represented using a "leaky" model, where vaccinated individuals have reduced susceptibility rather than complete immunity [10]. The population was segmented into three age groups—0-4, 5-19, and 20+ years—to align with the age categories reported by the CDC [8]. For age group i, the age-specific transitions among disease compartments as given by:

$$\frac{dS_{r,i}}{dt} = -\sum_{j\in A} \frac{\beta c_{ij} I_j(t) S_{1,i}(t)}{N_i} - \theta_i x_{contact,i}$$
$$\frac{dS_{n,i}}{dt} = -\theta_i (1 - x_{contact,i})$$
$$\frac{dV_{r,i}}{dt} = \theta_i x_{contact,i} - \sum_{j\in A} \frac{\beta c_{ij} I_j(t)(1-\epsilon) V_{1,i}(t)}{N_i}$$
$$\frac{dV_{n,i}}{dt} = \theta_i (1 - x_{contact,i})$$
$$\frac{dE_i}{dt} = \sum_{j\in A} \frac{\beta c_{ij} I_j(t)(S_{1,i}(t) + (1-\epsilon) V_{1,i}(t)))}{N_j} - \sigma E_i$$
$$\frac{dI_i}{dt} = \sigma E_i(t) - (IHR_i + \gamma_1) I_i(t)$$
$$\frac{dC_i}{dt} = \rho I_i$$
$$\frac{dH_i}{dt} = IHR_i I_i(t) - (HDR_i + \gamma_2) H_i(t)$$
$$\frac{dR_i}{dt} = \gamma_1 I_i(t) + \gamma_2 H_i(t)$$
$$\frac{dD_i}{dt} = HDR_i H_i(t)$$

where $S_{r,i}$,$S_{n,i}$,$V_{r,i}$,$V_{n,i}$,E_i,I_i, C_i,R_i,H_i,D_i are age-specific numbers of people who are in the “At-Risk” susceptible, “Non-Risk” susceptible, “At-Risk” vaccinated, “Non-Risk” vaccinated, exposed, infectious, reporting infectious, recovered, hospitalized and death compartments, respectively. β

© 2025 Bi K et al. *JAMA Health Forum*.

represents the time-dependent transmission rate; $c_{i,j}$ represents the number daily contacts between age group i to j; $x_{contact,i}$ represents the contactability proportion in the age group i; ϵ represents the vaccine efficacy, θ_i represents the daily vaccinated population in the age group i; σ represents the exposed to infectious rate; ρ represents the underreporting rate; IHR_i represents the infectious to hospitalization rate in age group i; HDR_irepresents the hospitalization to death rate in age group i; γ_1 and γ_2 represent the recovery rates from the infectious and hospitalizations. A is the set of the age groups, which include 0-4, 5-19, and 20+ years.

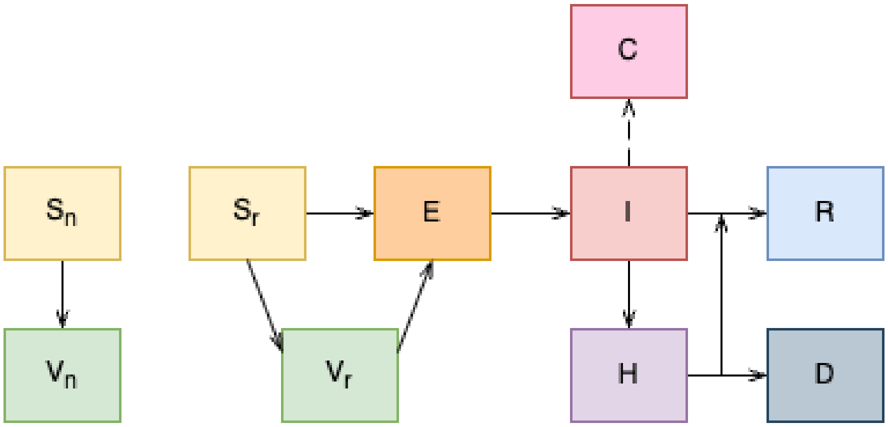

Figure S.1. Schematic representation of measles transmission model.

Model Assumption

To implement the counterfactual projections, we made the following assumptions:

Vaccine efficacy (VE): Vaccinated individuals experience reduced infection rates on a per-exposure basis, determined by the corresponding VE for one-dose or two-dose MMR vaccination[8].

Vaccine uptakes: Kindergarten MMR vaccination rates from the 2013–2014 to 2024–2025 school years[6] were used to estimate uptake for children aged 5 to 16 (for example., the 2024–2025 rate represents current 5-year-olds, 2023–2024 for current 6-year-olds, and so on.). For other age groups, uptake is assumed to equal the county-specific average kindergarten rate over this period.

Newly vaccinated population during simulation: Children aged 12–15 months are assumed to receive their first MMR dose[11], uniformly distributed throughout the year. Kindergarten-aged children received their second dose during the back-to-school period in August 2024 [12], so no second doses occur during the simulation period (January 20 to March 10, 2025).

Total Populations: Using the 2023 census data as the most recent source [7], we assumed the age distribution in 2025 is identical to that of 2023 for each county. The total population is considered stable during the simulation period, with natural births equaling natural deaths, and age progression is ignored given the period is less than one year.

Contact between age groups: Contacts between age groups follow the MOBS contact matrix[13], with both vaccinated and unvaccinated individuals within each age group having equal probability of contacting an infectious case.

© 2025 Bi K et al. *JAMA Health Forum*.

Initial States: The outbreak is seeded with a single infectious individual in the 5–19 age group, while the exposed, recovered, hospitalized, and deceased compartments are initialized at zero. For each county and age group, the vaccinated population is assigned based on reported vaccination coverage, and further divided into contactable ("at-risk") vaccinated individuals and non-contactable ("non-risk") vaccinated individuals according to the estimated contactability proportion. The susceptible population, defined as the total population minus the vaccinated population, is similarly split into "at-risk" and "non-risk" groups based on the same proportion.

Other assumptions: Asymptomatic and unreported measles cases are excluded due to limited data. All hospitalizations are assumed to occur among infectious individuals, and all deaths are assumed to arise from these hospitalizations.

Model Parameters

We present the parameter values and age-specific contact matrix in **Tables S.1** and **S.2**, respectively. The 3×3 contact matrix in **Table S.2** was aggregated from an 85×85 matrix provided by the MOBS Lab by consolidating ages into three groups: 0–4, 5–19, and 20+[13].

Table S.1. Parameter values for measles model

Parameter	Notation	Value	Reference
Transmission rate	β	Calibrated to case data	[4]
Contact between age group i and j	$c_{i,j}$	Contact matrix in **Table S.2**	[13]
Contactability proportion	$x_{contact,i}$	Calibrated to case data	[4]
Newly vaccinated population	θ_i	Derive from vaccine uptake	[9]
Vaccine efficacy	ϵ	93% for 0-4 in first dose<br>97% for 5+ in second dose	[8,11]
Exposed to infectious rate	σ	1/7-1/14 daily rates	[14]
Underreporting rate	ρ	Derive from literature	[15]

© 2025 Bi K et al. *JAMA Health Forum*.

Recovery rate from infectious	γ_1	1/4-1/7 daily rates	[16]
Recovery rate from hospitalizations	γ_2	1/5-1/6 daily rates	[17]
Infectious to Hospitalization rate in age group i	IHR_i	Derive from 29%, 13%, 17% hospitalized in age groups	[8]
Hospitalization death rate	HDR_i	Derive from 0.1-0.3%	[14]
Total population	N_i	Population distribution by age	[7]
Initial value for “At-Risk” Susceptible	$S_{r,i}(0)$	Derive from total population and vaccination	Assumed
Initial value for “Non-Risk” Susceptible	$S_{n,i}(0)$	Derive from total population and vaccination	Assumed
Initial value for “At-Risk” Vaccinated	$V_{r,i}(0)$	Derive from the total vaccination administration	[9]
Initial value for “Non-Risk” Vaccinated	$V_{n,i}(0)$	Derive from the total vaccination administration	[9]
Initial exposed	$E_i(0)$	0	Assumed
Initial Infectious	$I_i(0)$	0 in age group 0-4 and 20+; 1 in age group 5-19	Assumed
Initial recovered	$R_i(0)$	0	Assumed
Initial hospitalized	$H_i(0)$	0	Assumed

© 2025 Bi K et al. *JAMA Health Forum*.

Initial deceased	$D_i(0)$	0	Assumed

Equations for susceptible and vaccinated are as following:

$$S_{r,i}(0) = (N_i - V_{total,i})x_{contact,i}$$

$$S_{n,i}(0) = (N_i - V_{total,i})(1 - x_{contact,i})$$

$$V_{r,i}(0) = V_{total,i}x_{contact,i}$$

$$V_{n,i}(0) = V_{total,i}(1 - x_{contact,i})$$

Table S.2. Age-specific contact matrix in Texas

Age of contact\ age of be contacted	**0-4**	**5-19**	**20+**
**0-4**	1.4101	2.5282	4.91
**5-19**	0.9290	11.0091	6.0771
**20+**	0.4884	1.6387	8.8461

Model Calibration and Simulation

We calibrate age-specific transmission rates ($\beta_0, \beta_1, \beta_2$) and contactability proportions ($x_{contact}$) of the baseline model to the age-stratified cumulative reported cases in Gainescounty. The fitting period spanned from January 20, 2025, to Jun 10, 2025. Calibration was performed by minimizing the sum of squared errors between observed and model-simulated case counts, using Python's scipy.optimize.curve_fit function, which applies the Levenberg–Marquardt (damped least-squares) algorithm. All counterfactual scenarios subsequently adopt these baseline parameter estimates.

To account for uncertainty in epidemic dynamics, we conducted stochastic simulation using the age-structured compartmental model across multiple Texas counties. Each simulation run proceeds in discrete time steps (dt = 1 day) over a150-day period. During each run, transmission rates are perturbed by Gaussian noise with the approximate standard deviation estimated from fitting to reflect random fluctuations in contact-based transmission. In addition, key transition parameters including the incubation rate (σ), recovery rates (γ_1, γ_2), infectious-to-hospitalization rate (IHR), and hospitalization-to-death rate (HDR) are sampled from uniform distributions, while underreporting rate

© 2025 Bi K et al. *JAMA Health Forum*.

(ρ) is sampled from a truncated normal distribution at each time step to capture uncertainty in reporting and disease progression.

For each scenario projection, we run 200 stochastic simulations. We compute the projected measles cases, hospitalizations and deaths and summarize their evolving distributions using the 0.025, 0.50, and 0.975 quantiles for each day.

Scenarios

Baseline scenario: For each county, we use the reported MMR vaccination rates and introduce one infectious case (patient zero) on January 20, 2025, and apply the transmission rate calibrated from Gaines County.

Reduced vaccination uptake scenario: For each county, we assume MMR vaccination rates are 5% lower than the reported rates across all three age groups, and introduce one infectious case on January 20, 2025, and apply the transmission rate calibrated from Gaines County.

Increased vaccination uptake scenario: For each county, we assume MMR vaccination rates are 5% higher than the reported rates across all three age groups, and introduce one infectious case on January 20, 2025, and apply the transmission rate calibrated from Gaines County.

© 2025 Bi K et al. *JAMA Health Forum*.

eReferences

1. Conis E. Measles and the Modern History of Vaccination. Public Health Rep. 2019;134: 118–125.

2. Clemmons NS, Wallace GS, Patel M, Gastañaduy PA. Incidence of Measles in the United States, 2001-2015. JAMA. 2017;318: 1279–1281.

3. Measles Outbreak in Gaines County, Texas. [cited 7 Mar 2025]. Available: https://www.dshs.texas.gov/news-alerts/measles-outbreak-gaines-county-texas

4. News & Alerts. [cited 7 Mar 2025]. Available: https://www.dshs.texas.gov/news-alerts

5. Plotkin SA. Mass Vaccination: Global Aspects - Progress and Obstacles. Springer Science & Business Media; 2006.

6. [No title]. [cited 7 Mar 2025]. Available: https://public.tableau.com/app/profile/maria.tomasso/viz/measles_2025/Home

7. US Census Bureau. Census.gov | U.S. Census Bureau Homepage. 2025 [cited 9 Mar 2025]. Available: https://www.census.gov/en.html

8. CDC. Measles Cases and Outbreaks. In: Measles (Rubeola) [Internet]. 6 Mar 2025 [cited 7 Mar 2025]. Available: https://www.cdc.gov/measles/data-research/index.html

9. School Coverage. [cited 9 Mar 2025]. Available: https://www.dshs.texas.gov/immunizations/data/school/coverage

10. Lee DI, Nande A, Anderson TL, Levy MZ, Hill AL. Vaccine failure mode determines population-level impact of vaccination campaigns during epidemics. J R Soc Interface. 2025;22: 20240689.

11. Measles, Mumps, and Rubella (MMR) Vaccination. 19 Dec 2024 [cited 9 Mar 2025]. Available: https://www.cdc.gov/vaccines/vpd/mmr/public/index.html

12. Back-to-School. [cited 9 Mar 2025]. Available: https://www.dshs.texas.gov/immunizations/school/back-to-school

13. Mistry D, Litvinova M, Pastore Y Piontti A, Chinazzi M, Fumanelli L, Gomes MFC, et al. Inferring high-resolution human mixing patterns for disease modeling. Nat Commun. 2021;12: 323.

14. CDC. Measles Symptoms and Complications. In: Measles (Rubeola) [Internet]. 17 Jan 2025 [cited 9 Mar 2025]. Available: https://www.cdc.gov/measles/signs-symptoms/index.html

15. Yang W. Transmission dynamics of and insights from the 2018-2019 measles outbreak in New York City: A modeling study. Sci Adv. 2020;6: eaaz4037.

16. Measles. [cited 9 Mar 2025]. Available: https://www.who.int/news-room/fact-sheets/detail/measles

17. Measles. [cited 9 Mar 2025]. Available: https://www.who.int/news-room/fact-sheets/detail/measles

© 2025 Bi K et al. *JAMA Health Forum*.
