# Supplementary material for: Modeling the Impact of MMR Vaccination Strategies on Measles Outbreaks in Texas
Source: JAMA Health Forum. 2025 Sep 19;6(9):e253992. doi: 10.1001/jamahealthforum.2025.3992 (PMC12449715; doi:10.1001/jamahealthforum.2025.3992)
Supplement: Supplement 2. — Data Sharing Statement [file jamahealthforum-e253992-s002.pdf]

Data Sharing Statement

Bi. Modeling the Impact of MMR Vaccination Strategies on Measles Outbreaks in Texas. *JAMA Health Forum*. Published September 19, 2025. doi:10.1001/jamahealthforum.2025.3992

Data
Data available: Yes
Data types: Other (please specify)
Additional Information: Simulation results
How to access data: https://github.com/bikaiming93/measles_model
When available: With publication

Supporting Documents
Document types: Statistical/analytic code
How to access documents: https://github.com/bikaiming93/measles_model
When available: With publication

Additional Information
Who can access the data: anyone requesting the data
Types of analyses: for any purpose
Mechanisms of data availability: without investigator support
